# Cerebral Hemodynamic Alterations in Dialysis COVID-19 Survivors: A Transcranial Doppler Ultrasound Study on Intracranial Pressure Dynamics

**DOI:** 10.3390/kidneydial5020012

**Published:** 2025-04-03

**Authors:** José Lapeña-Motilva, Daniel Fouz-Ruiz, Mariano Ruiz-Ortiz, Eduardo Sanpedro-Murillo, Sara Gómez-Enjuto, Inés Hernando-Jimenez, Aida Frias-González, Andrea Soledad Suso, Evangelina Merida-Herrero, Julián Benito-León

**Affiliations:** 1 Department of Neurology, 12 de Octubre University Hospital, Av. de Córdoba, s/n, 28041 Madrid, Spain; 2 Department of Neurology, Severo Ochoa University Hospital, Av. de Orellana, s/n, 28914 Leganés, Spain; 3 Department of Nephrology, 12 de Octubre University Hospital, Av. de Córdoba, s/n, 28041 Madrid, Spain; 4 Department of Nephrology, Severo Ochoa University Hospital, Av. de Orellana, s/n, 28914 Leganés, Spain; 5 Instituto de Investigación Sanitaria Hospital 12 de Octubre (imas12), Av. de Córdoba, s/n, 28041 Madrid, Spain; 6 Centro de Investigación Biomédica en Red Sobre Enfermedades Neurodegenerativas (CIBERNED), C/Valderrebollo, 5, 28031 Madrid, Spain; 7 Department of Medicine, Complutense University, Pl. de Ramón y Cajal, s/n, 28040 Madrid, Spain

**Keywords:** SARS-CoV-2, hemodialysis, middle cerebral artery, pulsatility index, transcranial Doppler ultrasound, COVID-19

## Abstract

**Background:**

We observed a COVID-19 survivor with a ventriculoperitoneal shunt who developed increased intracranial pressure during hemodialysis. We hypothesized that post-SARS-CoV-2 infection, patients may have altered cerebral perfusion pressure regulation in response to intracranial pressure changes.

**Methods:**

From April to July 2021, we recruited dialysis patients with prior COVID-19 from two Madrid nephrology departments. We also recruited age- and sex-matched dialysis patients without prior SARS-CoV-2 infection. Transcranial Doppler ultrasound was used to measure the middle cerebral artery velocity before dialysis and 30, 60, and 90 min after the initiation of dialysis.

**Results:**

The final sample included 37 patients (16 post-COVID-19 and 21 without). The COVID-19 survivors showed a significant pulsatility index increase between 30 and 60 min compared to those without COVID-19. They also had lower heart rates.

**Conclusions:**

We propose two mechanisms: an increase in intracranial pressure or a decreased arterial elasticity. A lower heart rate was also observed in the COVID-19 survivors. This study highlights SARS-CoV-2’s multifaceted effects, including potential long-term vascular and cerebral repercussions.

## 1. Introduction

Severe Acute Respiratory Syndrome Coronavirus 2 (SARS-CoV-2) infection poses significant challenges, affecting not only the respiratory system but also the endothelium across various organs due to its tropism for angiotensin-converting enzyme 2 (ACE-2) [1,2]. Direct infection of endothelial cells leads to morphological changes, including swelling and cell death [3]. This can result in endotheliitis, contributing to endothelial dysfunction, which impairs normal functions such as coagulation and blood flow regulation [4]. Endothelial dysfunction is particularly critical in COVID-19 pathogenesis, especially in patients with pre-existing comorbidities [5]. Post-mortem studies reveal endotheliitis and damage in various organs [6], while elevated endothelial dysfunction markers have been observed in COVID-19 patients [7]. Furthermore, long-term endothelial dysfunction may contribute to “Long COVID” symptoms and increase the risk of cardiovascular diseases [8].

After overseeing the case of a COVID-19 survivor with a ventriculoperitoneal bypass valve who developed increased intracranial pressure during hemodialysis [9], we hypothesized that, following an acute SARS-CoV-2 infection, patients could experience alterations in the regulation of cerebral perfusion pressure in response to changes in intracranial pressure. Notably, studies have shown that the pulsatility index (PI) can increase in patients post-COVID-19, with one study reporting a significantly higher PI in the middle cerebral artery (MCA) after a breath-holding test in COVID-19 patients compared to controls, suggesting impaired cerebral vascular reactivity and potential endothelial dysfunction [10]. Additionally, some patients have been reported to develop intracranial hypertension after COVID-19 [11]. To address our hypothesis, we evaluated changes in the PI in the MCA using transcranial Doppler ultrasound as a marker of intracranial pressure variation before and during hemodialysis.

## 2. Material and Methods

### 2.1. Design

From April 2021 to July 2021, we consecutively recruited 25 dialysis patients who had COVID-19 from the databases of two nephrology departments in Madrid (Spain). We chose dialysis patients due to its high hemodynamic impact and reproducibility, independent of patient collaboration.

COVID-19 diagnosis was based on clinical presentation and having a positive PCR test or serology before vaccination. The measurements were taken after vaccination, and patients who were infected during this period were excluded.

The sample consisted of 25 patients (three women; mean age = 67.5 years) and 24 controls (nine women; mean age = 66.8 years). Transplanted patients, those who had previously suffered an ischemic stroke or had stenosis of one of the carotid arteries greater than 70%, and those who, due to their intrinsic characteristics, could not consent or did not sign the informed consent form were excluded.

### 2.2. Measurement

Measurements of peak systolic velocity (PSV) and final diastolic velocity (FDV) were performed in the middle cerebral artery (left or right, depending on anatomical characteristics and the distribution of machines in the room) before and 30, 60, and 90 min after starting dialysis. Experienced neurologists performed all measurements. The variables additionally recorded were the PI, resistance index (RI), estimated cerebral perfusion pressure (eCPP), and mean blood pressure (MBP), according to the formulas present in Table 1 [12].

Analyses were performed before and after dialysis, in which blood count was requested with hematocrit, carbon dioxide, bicarbonate, ionic calcium, creatinine, sodium, potassium, calcium, magnesium, phosphorus, and urea. Patients who presented erroneous or empty values due to errors in the extraction or processing of the sample were not excluded from the analysis.

### 2.3. Statistical Analyses

The analyses were performed in Python 3.9.7 using the following packages: pandas 1.3.4, matplotlib 3.4.3, numpy 1.20.3, seaborn 0.11.2, scipy 1.7.1, tableone 0.7.10 [13], and statsmodels 0.12.2.

Differences in PI, RI, eCPP, heart rate (HR), systolic blood pressure (SBP), diastolic blood pressure (DBP), and MBP between intakes 1 and 2, 2 and 3, and 3 and 4 were calculated. Parametric and non-parametric tests were used as appropriate. Furthermore, secondary multivariate analyses were performed using ordinary least squares (OLS) linear regression.

A secondary analysis was performed, segmenting the patients into those who had an increase in PI during the first hour of dialysis (difference between measurements at 0 and 30 min or 30 and 60 min) vs. those who did not experience an increase.

### 2.4. Ethical Approval and Consent to Participate

The ethical standards committees approved all procedures on human experimentation at the 12 de Octubre University Hospital (Madrid) [Approval Code: 21/158, Date: 23 March 2021]. Written (signed) informed consent was obtained from all enrollees.

### 2.5. Data Availability Statement

The data generated or analyzed during this study are available from the corresponding author upon reasonable request.

## 3. Results

Thirteen patients were excluded due to measurements delayed by over 10 min. The final sample consisted of 37 patients (Table 2). Groups differed only in magnesium levels before the start of dialysis and their variation (Table 3).

**Figure 1.**
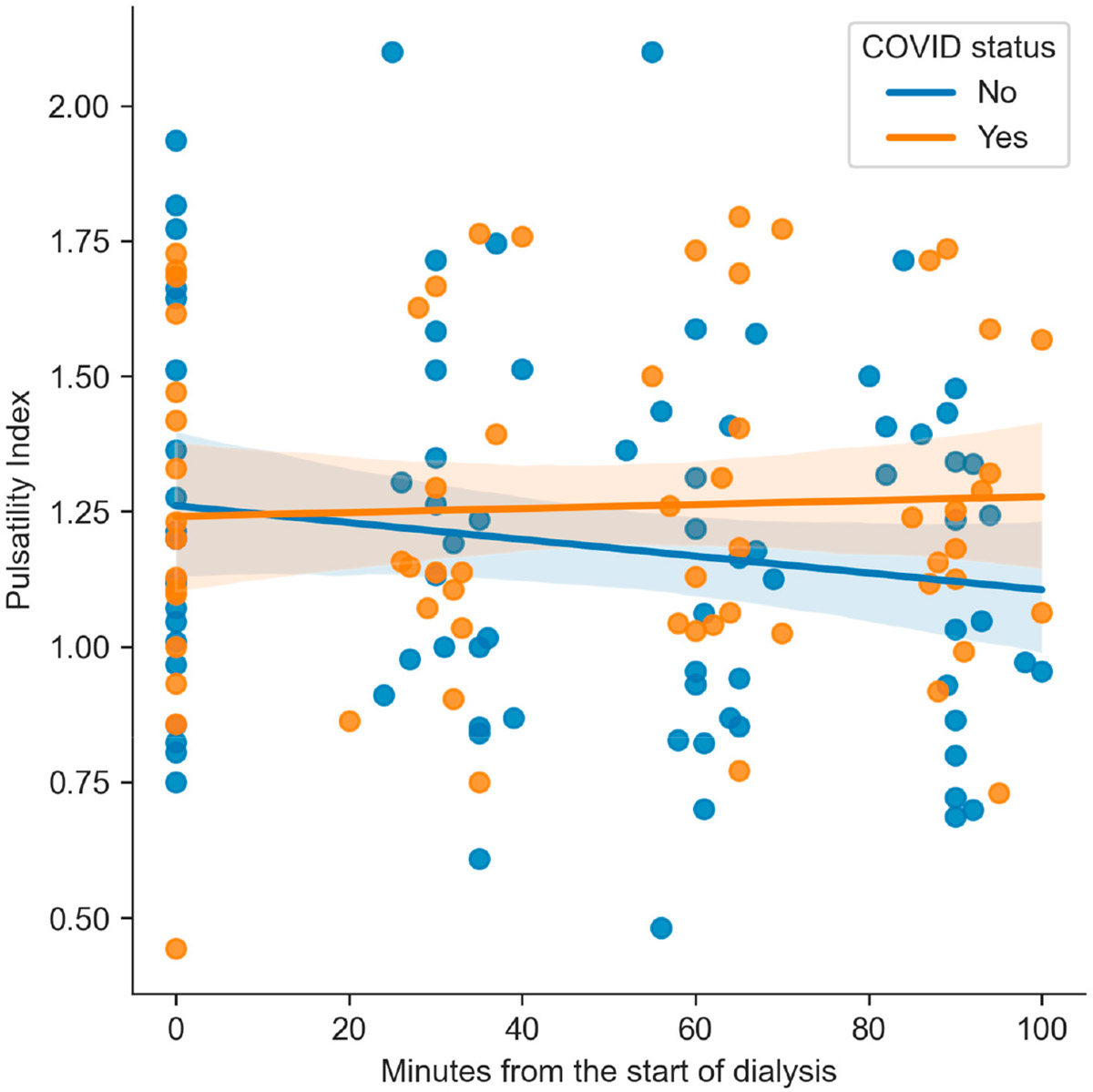
The pulsatility index depending on the COVID-19 status in each measurement and the linear trend.

**Figure 2.**
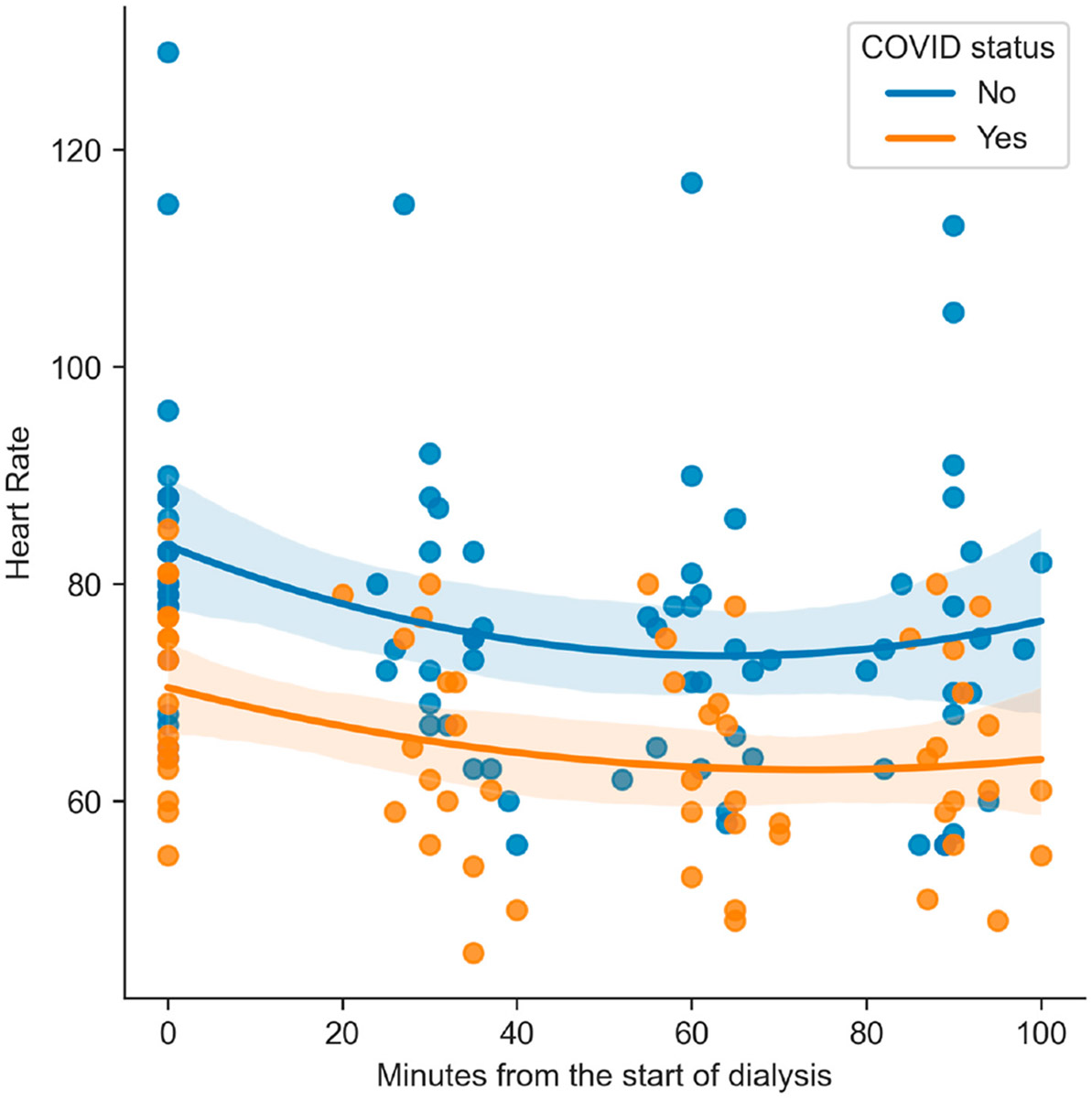
Heart rate depending on the COVID-19 status in each measurement and the quadratic trend.

No significant differences were found between the groups for PI (Figure 1), RI, SBP, MBP, DBP, or eCPP. However, HR differed significantly between patients with and without COVID-19 at all measured time points. The mean HR was lower in the COVID group at the baseline (83.19 (SD 15.5) bpm vs. 70.31 (SD 8.9) bpm, *p* = 0.005), at 30 min (75.71 (SD 13.1) bpm vs. 64.56 (SD 10.4) bpm, *p* = 0.008), at 60 min (74.29 (SD 13) bpm vs. 63.38 (SD 9.6) bpm, *p* = 0.008), and at 90 min (74.81 (SD 15.5) bpm vs. 64.06 (SD 9.4) bpm, *p* = 0.019). These results indicate a consistently lower HR in patients who had suffered from COVID-19 (Figure 2).

**Figure 3.**
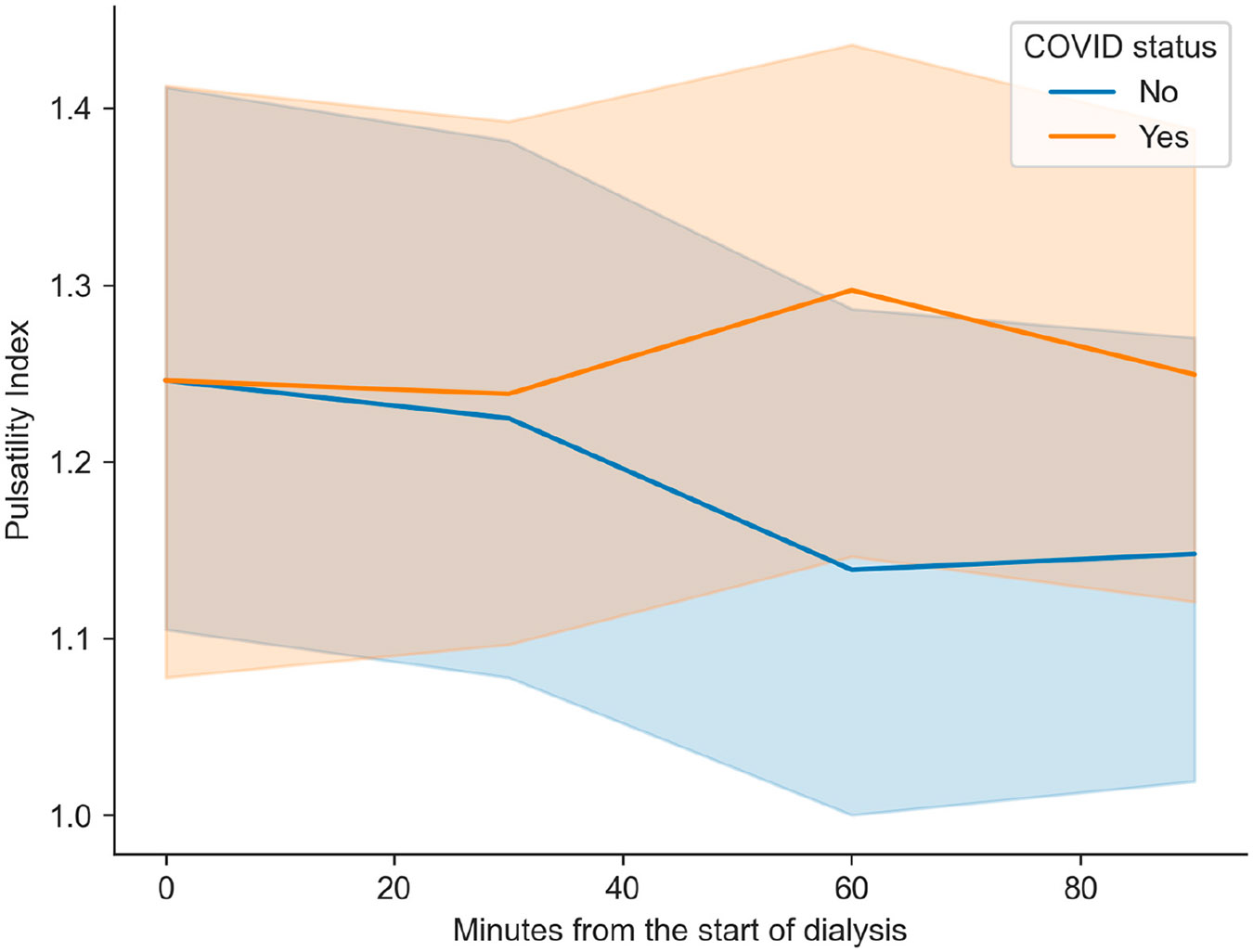
Pulsatility index depending on COVID-19 status and each measurement. The mean and 95% confidence interval are shown for each measurement.

**Figure 4.**
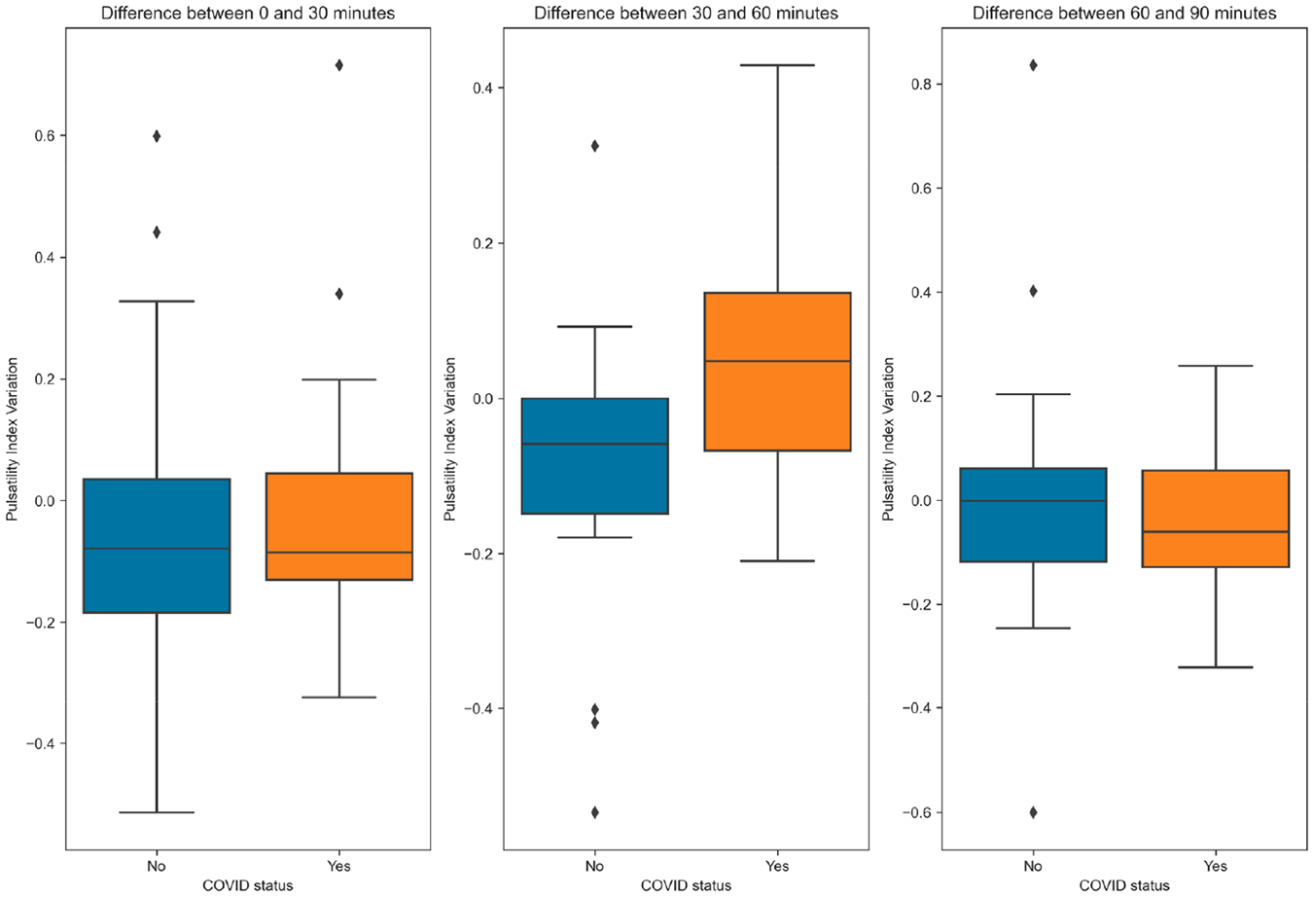
Boxplot showing the variation in the pulsatility index between one measurement and the next. Segments are based on COVID-19 status.

No significant differences were observed between the groups for DBP, SBP, HR, or eCPP when comparing the inter-measurement variations. However, the PI variation differed significantly between the groups at 30 min and 60 min between the non-COVID-19 and COVID-19 patients (−0.1 (SD 0.2) vs. 0.1 (SD 0.2), *p* = 0.023). Furthermore, the FDV (1.7 (SD 6.1) cm/s vs. −3.8 (SD 7.2) cm/s, *p* = 0.022) shows differences between the controls and patients, respectively. No significant difference in PI or FDV variation was observed between the other measurements (Figures 3 and 4)

The multivariate linear regression showed that COVID-19 status (*p* = 0.032) and age (*p* = 0.016) independently predicted PI variation between the second and third assessments. The creatinine level difference also showed a significant negative relationship (*p* = 0.020) (Table 4).

The patients were segmented by the PI increase during the first hour of dialysis. No significant differences were observed in COVID-19 infection percentage, personal history, or other baseline characteristics. However, a trend towards a lower baseline level of creatinine was noted (8.7 (SD 1.8) mg/dL vs. 7.6 (SD 1.5) mg/dL, *p* = 0.077) between the patients without an increase in the PI and those with an increase, respectively. The latter group also had a lower initial PI (1.4 (SD 0.3) vs. 1.2 (SD 0.3), *p* = 0.044) and a higher initial MBP (105.5 (SD 23.7) mmHg vs. 121.8 (SD 19.3) mmHg, *p* = 0.045). The initial HR did not differ significantly (*p* = 0.079).

In the measurements for which an increase was observed, there were no significant differences in PSV (−1.5 (SD 15.8) cm/s vs. −0.7 (SD 18.5) cm/s, *p* = 0.858) between the non-incremental and incremental measures, respectively, but notable differences were found in the FDV (2.8 (SD 5.6) cm/s vs. −4.4 (SD 7.2) cm/s, *p* < 0.001).

## 4. Discussion

This study assessed the association between variations in brain flow during dialysis and a previous SARS-CoV-2 infection. Despite the differences being small, we found a significant increase in the PI, which is associated with increased intracranial pressure [11,14], between the second and third measurements in patients who had COVID-19 compared to those who did not, suggesting pathological cerebral hemodynamic responses in the first ones.

Pressure changes in the central nervous system have been previously described in dialysis patients, corresponding to the imbalance syndrome in its most severe expression [15]. This syndrome consists of the appearance of neurological symptoms related to cerebral edema caused by increased intracranial pressure [15].

There are two possible pathophysiological mechanisms of the increase in PI in the patients who had COVID-19.

First, the existence of a dysfunction of the BBB could potentially contribute to an increase in intracranial pressure in these patients; however, this remains a speculative hypothesis that requires further investigation to establish a definitive link. The relationship between SARS-CoV-2 and BBB dysfunction is complex and not fully understood. Evidence suggests that the virus may invade the brain and alter BBB permeability [16], allowing serum components to enter the central nervous system. This increased permeability has been observed during the acute phase of infection and in patients experiencing persistent cognitive symptoms, often referred to as “brain fog” [17].

Several mechanisms may disrupt the BBB, including coagulopathy, the disruption of tight junctions, and hypoxia-induced permeability changes [16]. Additionally, neuroimaging studies have shown brain abnormalities in COVID-19 patients, and the presence of viral RNA in cerebrospinal fluid indicates the potential for neuroinvasion [16]. Elevated levels of inflammatory markers, such as S100β, have also been associated with BBB dysfunction in patients with cognitive impairment [17]. However, the persistence of these alterations and their clinical implications remain uncertain, necessitating further research to clarify the underlying mechanisms and their impact on neurological outcomes.

Imbalance syndrome is thought to be caused by loss of osmotic balance during dialysis since some solutes, such as urea, take longer to be cleansed from cerebrospinal fluid than from blood [18]. This syndrome is favored by pathologies that increase the permeability of the BBB [19], but its role in urea transport is unclear [20]. The role of the BBB in cerebral edema due to multiple causes (traumatic, oncological, and others [21-23]) has also been widely studied, so it seems that the BBB may have a fundamental role in the management of solutes and water and, therefore, of intracranial pressure during dialysis.

Second, a decrease in the elasticity of the arteries may lead to an increase in the PI to maintain the CPP. This decrease in elasticity could be caused by endothelial dysfunction. SARS-CoV-2 has a tropism for both endothelial [24] and epithelial cells in the choroid plexuses [25], which may contribute to a chronic alteration of the BBB. Likewise, there is an inflammatory endothelial dysregulation in COVID-19 patients during the acute phase, characterized by elevated levels of biomarkers of endothelial dysfunction, such as endothelin-1 (ET-1), which could produce long-term vascular involvement [2,26-28].

This endothelial dysfunction manifests as reduced flow-mediated vasodilation in peripheral arteries and decreased cerebral vascular reactivity [27]. Additionally, the hypercoagulable state associated with endothelial dysfunction can lead to the formation of microthrombi and hemorrhages [28]. Several vascular diseases, including arterial hypertension, obesity, diabetes mellitus, and coronary heart disease, share this pathophysiological mechanism. It is known that patients with more vascular risk factors have higher PI values in the middle cerebral artery [29], causing a decrease in the elasticity of the arteries.

From the secondary analyses we carried out, a significantly lower HR was detected in the group of patients who had COVID-19 compared with the group that had not, but there were no differences in the variation in these during hemodialysis. Evidence concerning the effects of SARS-CoV-2 on HR is contradictory, including studies depicting both a decline [30] and an increase [31] in HR at rest. Postural orthostatic tachycardia syndrome cases have been described in post-COVID-19 patients [32,33], which, although they do not share the same assessment criteria, would go against our findings. More studies are needed to elucidate this correlation.

Finally, in dialysis patients, independent from whether they had COVID-19 or not, in whom we detected an increase in the PI in the measurement at 30 or 60 min, we found that the parameters most related to this increment were having a greater MBP, a lower PI in the basal measurement, and an increase in the MBP during the repeated measurements. Likewise, an increase in FDV was detected in the third measurement in the group that did not present an increase in PI without changes in the PSV. All of this could reflect a decrease in peripheral resistance in the group without an increase in PI or an increase in the one group with an increase in PI. Despite these MCA neurosonographic changes, there was no variation in MBP or HR in these patients, so intracranial vascular alterations should cause differences in these values.

There was a statistical trend in creatinine levels in these patients, with lower values at baseline in patients with an increased PI during the first or second measurement. This may not directly correlate with the values found since it is used as a nutritional parameter in dialysis patients [34].

Our results in the multivariate analysis showed a relevant role of the starting MBP in these differences, but there were no differences in this measurement between the evaluations, making us think that blood pressure is not the only influential factor. This could be explained by the increased intracranial pressure in these patients^17^, which, even if they were asymptomatic, could increase the PI.

These results are consistent with those described by Marcic et al. [27], who found evidence of chronic endothelial dysfunction in post-COVID-19 patients assessed through transcranial Doppler ultrasound 300 days post-infection. In their study, the reduced breath-holding index reflected impaired cerebral vasoreactivity. These authors proposed that the alteration in cerebral vasoreactivity may be mediated by persistent endothelial inflammation and a hypercoagulation state in these patients [27].

Our study should be interpreted considering several limitations. First, our study did not reach the pre-estimated *n* value. Even so, we believe this did not affect the outcome of the results, as the *n* was predicted considering the differences in PI between patients with and without IH. Second, we did not record symptoms that could impact the patients’ day-to-day lives, so we could not correlate the increase in PI with possible symptomatology. We did not obtain data on the severity of COVID-19 since most of the infections were presented during the first wave, and there were no reliable records; nevertheless, most patients presented mild cases that did not require hospitalization or were diagnosed through antibody testing after asymptomatic infection. Third, dialysis patients do not represent the general population because they have significant comorbidity, especially in arterial pathology. Fourth, the transcranial Doppler ultrasound is an operator-dependent test with a non-depreciable random error, which we have tried to minimize by having neurologists with experience perform the measurements. Additionally, we acknowledge that continuous monitoring would be more appropriate for assessing changes; however, this technique was not available in either of the hospitals where the study was conducted.

## 5. Conclusions

Our exploratory study aimed to investigate alterations in the regulation of cerebral hemodynamics in dialysis patients who have had COVID-19. While we observed trends suggesting a lower compensation range for potential changes in intracranial pressure, these findings should be interpreted with caution, as they are not directly linked to clinical outcomes. These results are consistent with previous studies, such as that of Marcic et al. [27], which highlight the prolonged impact of SARS-CoV-2 on cerebral vasoreactivity.

Additionally, we detected a lower HR in this group of patients; however, we did not identify a clear pathophysiological explanation for this observation. Further studies are warranted in non-dialysis post-COVID-19 patients to mitigate potential confounding factors related to chronic arterial pathology in this population.

## Figures and Tables

**Table 1.** Formulas used for the calculation of variables. PSV: peak systolic velocity; FDV: final diastolic velocity; MV: mean velocity; MBP: mean blood pressure; DBP: diastolic blood pressure; SBP: systolic blood pressure.

Parameter	Formula
Pulsatility index	$\frac{PSV-FDV}{MV}$
Resistance index	$\frac{PSV-FDV}{PSV}$
Estimated cerebral perfusion pressure (mmHg)	$\frac{MBP*FDV}{MV}+14$
Mean blood pressure (mmHg)	$\frac{2*DBP+SBP}{3}$

**Table 2.** Comparison of clinical characteristics between non-COVID-19 patients and COVID-19 survivors. The table presents the number of participants, demographic information, comorbidities, laboratory values, and their respective *p*-values. ^a^ Chi-square test, ^b^ Fisher’s exact test, ^c^ Student’s *t*-test.

Variable	Non-COVID-19	COVID-19 Survivors	*p*-Value
Number of participants	21	16	
Men (*n*, %)	12 (57.1%)	13 (81.2%)	0.231 ^a^
Women (*n*, %)	9 (42.9%)	3 (20.0%)	
Age (mean ± SD)	66.8 ± 11.9 years	67.5 ± 15.1 years	0.869 ^c^
Hemodialysis duration (mean ± SD)	60.2 ± 72.9 months	98.2 ± 135.0 months	0.322 ^c^
Time since COVID-19 infection (mean ± SD)	-	13.3 ± 4.3 months	-
Arterial hypertension (*n*, %)	19 (90.5%)	15 (93.8%)	1.000 ^b^
Dyslipidemia (*n*, %)	14 (70.0%)	7 (43.8%)	0.212 ^a^
Diabetes mellitus (*n*, %)	10 (47.6%)	4 (25.0%)	0.288 ^a^
Atrial fibrillation (*n*, %)	6 (28.6%)	3 (18.8%)	0.702 ^b^
Heart failure (*n*, %)	6 (28.6%)	3 (18.8%)	0.702 ^b^
B-blockers or diltiazem use	6 (28.6%)	2 (12.5%)	0.423 ^b^
Limb ischemia (*n*, %)	5 (20.8%)	4 (15.4%)	0.721 ^b^
COPD (*n*, %)	1 (4.8%)	2 (5.4%)	1.000 ^b^
Cancer (*n*, %)	5 (23.8%)	6 (16.2%)	0.206 ^b^
Liver disease (*n*, %)	5 (23.8%)	8 (22.2%)	1.000 ^b^
Autoimmune diseases (*n*, %)	3 (14.3%)	4 (10.8%)	0.618 ^b^
Ischemic heart disease (*n*, %)	4 (19.0%)	6 (16.2%)	0.680 ^b^
Stroke (*n*, %)	1 (4.8%)	2 (5.4%)	1.000 ^b^
Intracranial interventions (*n*, %)	0 (0.0%)	0 (0.0%)	1.000 ^b^
Intracranial hypertension (*n*, %)	0 (0.0%)	0 (0.0%)	1.000 ^b^
Intracranial hypotension (*n*, %)	0 (0.0%)	0 (0.0%)	1.000 ^b^
Tobacco use (*n*, %)	6 (31.6%)	10 (32.3%)	1.000 ^b^

**Table 3.** Comparison of dialysis parameters between non-COVID-19 patients and COVID-19 survivors. The table presents the mean values and standard deviations (SDs) for dialysis time, ultrafiltration volume, and various bath compositions, along with the corresponding *p*-values for each comparison. ^c^ Student’s *t*-test, ^d^ Kruskal–Wallis.

Variable	Non-COVID-19	COVID-19 Survivors	*p*-Value
Dialysis time (mean ± SD)	245.7 ± 13.2 min	244.8 ± 13.9 min	0.624 ^c^
Ultrafiltration volume (mean ± SD)	2493.9 ± 864.5 mL	2535.2 ± 771.4 mL	0.751 ^c^
QB (median [IQR])	350.0 [350.0, 400.0] mL/min	350.0 [350.0, 385.0] mL/min	0.588 ^d^
Sodium bath (mean ± SD)	137.4 ± 1.0 mEq/L	137.4 ± 1.2 mEq/L	0.794 ^c^
Potassium bath (median [IQR])	2.0 [2.0, 2.0] mEq/L	2.0 [2.0, 2.0] mEq/L	0.329 ^d^
Calcium bath (mean ± SD)	1.6 ± 0.1 mEq/L	1.6 ± 0.1 mEq/L	0.160 ^c^
Bicarbonate bath (mean ± SD)	33.2 ± 2.7 mEq/L	33.4 ± 2.5 mEq/L	0.555 ^c^
Glucose bath (median [IQR])	1.0 [1.0, 5.5] g/dL	1.0 [1.0, 5.5] g/dL	0.869 ^d^
Creatinine (mean ± SD)	7.8 ± 1.7 mg/dL	8.2 ± 1.7 mg/dL	0.458 ^c^
Difference in creatinine (mean ± SD)	−5.7 ± 1.4 mg/dL	−5.5 ± 1.9 mg/dL	0.744 ^c^
Hematocrit (mean ± SD)	34.9 ± 3.7%	33.7 ± 6.4%	0.503 ^c^
Difference in hematocrit (mean ± SD)	3.9 ± 2.7%	4.5 ± 7.2%	0.751 ^c^
Carbon dioxide (mean ± SD)	39.7 ± 5.1 mmHg	40.5 ± 6.0 mmHg	0.676 ^c^
Difference in carbon dioxide (mean ± SD)	0.9 ± 2.8 mmHg	1.6 ± 4.1 mmHg	0.572 ^c^
Sodium (mean ± SD)	139.0 ± 2.3 mEq/L	139.8 ± 3.2 mEq/L	0.436 ^c^
Difference in sodium (mean ± SD)	0.1 ± 2.9 mEq/L	−9.6 ± 34.7 mEq/L	0.297 ^c^
Potassium (mean ± SD)	5.0 ± 0.6 mEq/L	5.5 ± 0.8 mEq/L	0.051 ^c^
Difference in potassium (mean ± SD)	−1.6 ± 0.6 mEq/L	6.3 ± 33.4 mEq/L	0.355 ^c^
Calcium (mean ± SD)	8.9 ± 0.6 mg/dL	9.0 ± 0.4 mg/dL	0.781 ^c^
Difference in calcium (mean ± SD)	1.1 ± 0.7 mg/dL	0.7 ± 1.7 mg/dL	0.340 ^c^
Magnesium (mean ± SD)	2.1 ± 0.3 mg/dL	2.4 ± 0.4 mg/dL	0.023 ^c^
Difference in magnesium (mean ± SD)	−0.2 ± 0.2 mg/dL	−0.5 ± 0.4 mg/dL	0.016 ^c^

**Table 4.** Results of the multivariate linear regression analysis for the difference in PI between the second and third assessments. The table presents the coefficients, standard errors, *t*-values, *p*-values, and 95% confidence intervals for each variable included in the model.

Variable	Coefficient	Std. Error	*t*	*p*-Value
COVID-19	0.1481	0.065	2.273	0.032
Age	0.0041	0.002	2.583	0.016
Hemodialysis duration	0.0005	0.000	1.303	0.205
Sex	−0.1263	0.067	−1.877	0.073
DBP difference	−0.0010	0.003	−0.291	0.773
HR difference	−0.0030	0.009	−0.352	0.728
Creatinine difference	−0.0388	0.016	−2.490	0.020

## Data Availability

The datasets generated and/or analyzed during the current study are available from the corresponding author on reasonable request.

## Abbreviations

SARS-CoV-2: Severe Acute Respiratory Syndrome Coronavirus 2
ACE-2: angiotensin-converting enzyme 2
COVID-19: coronavirus disease 2019
PI: pulsatility index
IH: intracranial hypertension
PSV: peak systolic velocity
FDV: final diastolic velocity
RI: resistance index
CPPe: estimated cerebral perfusion pressure
MBP: mean blood pressure
HR: heart rate
SBP: systolic blood pressure
DBP: diastolic blood pressure
OLS: ordinary least squares
BBB: blood-brain barrier
